# Overview and challenges of machine translation for contextually appropriate translations

**DOI:** 10.1016/j.isci.2024.110878

**Published:** 2024-09-12

**Authors:** Palanichamy Naveen, Pavel Trojovský

**Affiliations:** 1 Department of Mathematics, Faculty of Science, University of Hradec Králové, Rokitanského 62, Hradec Králové, Czech Republic

**Keywords:** Engineering, Machine, Social sciences

## Abstract

Machine translation facilitates cross-linguistic communication by converting text between languages. However, producing contextually accurate translations remains a challenge. This review explores the difficulties in achieving such accuracy, particularly in capturing contextual information, disambiguating polysemous words, and handling idiomatic expressions, cultural nuances, and domain-specific terms. The article emphasizes the importance of maintaining grammatical correctness and syntactic coherence while preserving the cultural context of the source text. It also addresses challenges related to complex sentence structures and grammatical transformations. The broader significance lies in enhancing machine translation systems to better break language barriers, foster multicultural understanding, and support global collaboration.

## Graphical abstract

**Figure:**
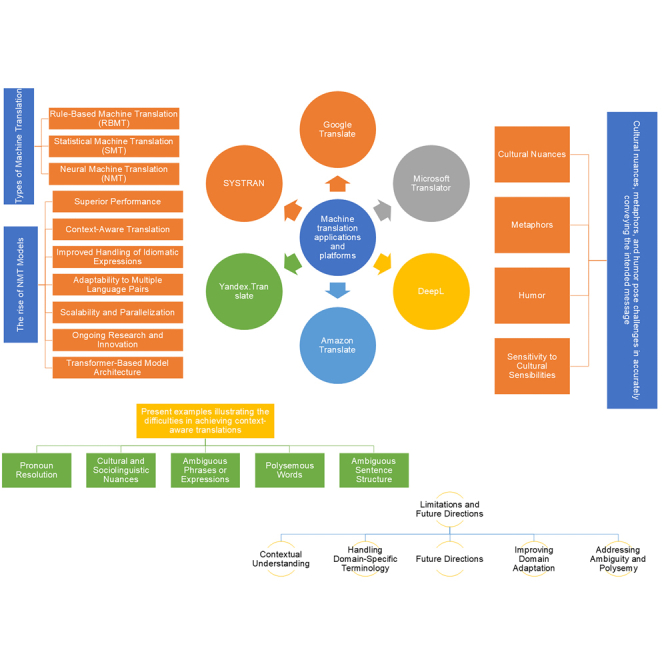

## Introduction

The field of machine translation has evolved rapidly, with numerous significant contributions that have shaped its current state. Recent advancements have focused on improving the accuracy, efficiency, and contextual understanding of machine translation models. Ranathunga et al. (2023) conducted a comprehensive survey on neural machine translation for low-resource languages, highlighting the challenges and potential solutions for improving translation quality in under-resourced languages.^1^ Dabre et al. (2020) reviewed multilingual neural machine translation techniques, providing insights into developing models capable of handling multiple languages simultaneously.^2^ Bao et al. (2023) explored non-autoregressive document-level machine translation, presenting innovative approaches to enhance translation efficiency and accuracy.^3^ Despite these advancements, several research gaps remain. One major gap is the handling of idiomatic expressions and cultural nuances, which are often lost in translation due to the lack of contextual understanding.^4^^,^^5^ Another significant gap is the translation of domain-specific terminology, which requires specialized knowledge and contextual awareness.^6^ This review article addresses these gaps by providing a detailed analysis of current machine translation models and proposing solutions to improve the handling of context, idiomatic expressions, and domain-specific terminology. By enriching the literature review with recent articles and critically discussing the research gaps, we aim to provide a comprehensive overview of the field and highlight the contributions of our work.

This article aims to provide an overview of machine translation and delve into the challenges faced in generating contextually appropriate translations. It explores various aspects of machine translation that contribute to the complexity of achieving accurate and contextually relevant translations. One of the primary challenges is capturing and utilizing contextual information from the source text. Context encompasses not only the immediate surrounding words but also the broader discourse and cultural background. Disambiguating polysemous words, handling idiomatic expressions, understanding cultural nuances, and resolving referential ambiguity are all intricately tied to context. Maintaining grammatical correctness and syntactic coherence is another significant challenge. Different languages exhibit variations in syntax and grammar structures, making it difficult to generate translations that adhere to the target language’s grammatical rules while preserving the source text’s intended meaning. Complex sentence structures, such as subordination and coordination, pose additional difficulties in ensuring the translated text flows naturally and maintains coherence.^7^

Accurate translation of technical, scientific, or domain-specific terms is yet another challenge. These terms often have specific meanings and connotations within their respective fields, requiring a deep understanding of the subject matter to generate accurate translations. The context in which these terms appear plays a crucial role in determining the most appropriate translation. The preservation of cultural and contextual aspects is vital in machine translation. Translating idiomatic expressions, metaphors, humor, and other culturally specific elements is challenging, as their meaning may not be directly translatable. Capturing cultural nuances and maintaining cultural sensitivity in translations are essential to convey the intended message accurately.^8^^,^^9^

By addressing these challenges, machine translation can break language barriers, foster multicultural understanding, and enable global collaboration. It opens up new opportunities for communication, information exchange, and cooperation across diverse linguistic communities. Thus, achieving contextually appropriate translations in machine translation systems is a complex task that requires addressing various challenges. Capturing and utilizing contextual information, handling idiomatic expressions and cultural nuances, maintaining grammatical correctness and syntactic coherence, and accurately translating technical or domain-specific terms are key areas that demand attention. By addressing these challenges, machine translation can pave the way for effective cross-lingual communication and contribute to advancing global collaboration and understanding.

## Machine translation – an overview

### Definition of machine translation

Machine translation refers to the automated process of transforming text or speech from one language, known as the source language, into another language, known as the target language. It involves utilizing computational models and algorithms to analyze and understand the input in the source language and generate an equivalent representation in the target language. Machine translation aims to facilitate communication and understanding between individuals who speak different languages, enabling the exchange of information, ideas, and knowledge across linguistic boundaries. Various techniques and approaches, including rule-based systems, statistical models, and neural networks, are employed in machine translation to achieve accurate and contextually appropriate translations.^10^

### Types of machine translation

**Figure 1:**
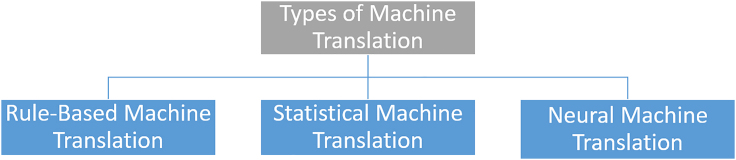
Types of machine translation

Machine translation approaches can be broadly classified into three main types: rule-based machine translation, statistical machine translation, and neural machine translation (NMT), as represented in Figure 1. Each approach utilizes different techniques and methodologies to accomplish the task of translation.

#### Rule-based machine translation

Rule-based machine translation relies on explicit linguistic rules and dictionaries to perform translations. Linguistic experts manually create these rules, which include syntactic and grammatical patterns, vocabulary mappings, and transformation rules. RBMT systems analyze the source text, apply the predefined rules, and generate the corresponding translation in the target language. Advantages of RBMT include control over grammar and syntax, making it suitable for certain domains and languages. However, RBMT has limitations in handling idiomatic expressions, language variations, and extensive lexical resources.^11^^,^^12^

#### Statistical machine translation

Statistical machine translation involves the use of statistical models and algorithms to translate text. SMT systems are trained on large parallel corpora that consist of aligned sentences in the source and target languages. The models learn statistical patterns and relationships between the source and target languages during training. The translation process involves estimating the most probable translation based on the learned statistical patterns. SMT was a dominant approach before the rise of NMT and allowed for the effective handling of complex language structures and variations. However, SMT often requires extensive linguistic resources and struggles with capturing long-range dependencies.^13^^,^^14^

#### Neural machine translation

Neural machine translation represents a paradigm shift in machine translation, leveraging artificial neural networks to model the translation process. NMT models, particularly those based on transformer architectures, have gained significant popularity due to their impressive performance. These models consist of encoder and decoder components, with attention mechanisms enabling the capture of contextual dependencies. NMT models learn to generate translations end-to-end without relying on explicit rule-based or statistical alignments. The advantage of NMT is its ability to handle long-range dependencies, capture context, and generate fluent translations. NMT models can effectively handle idiomatic expressions and have shown promising results across various language pairs.^15^^,^^16^

**Figure 2:**
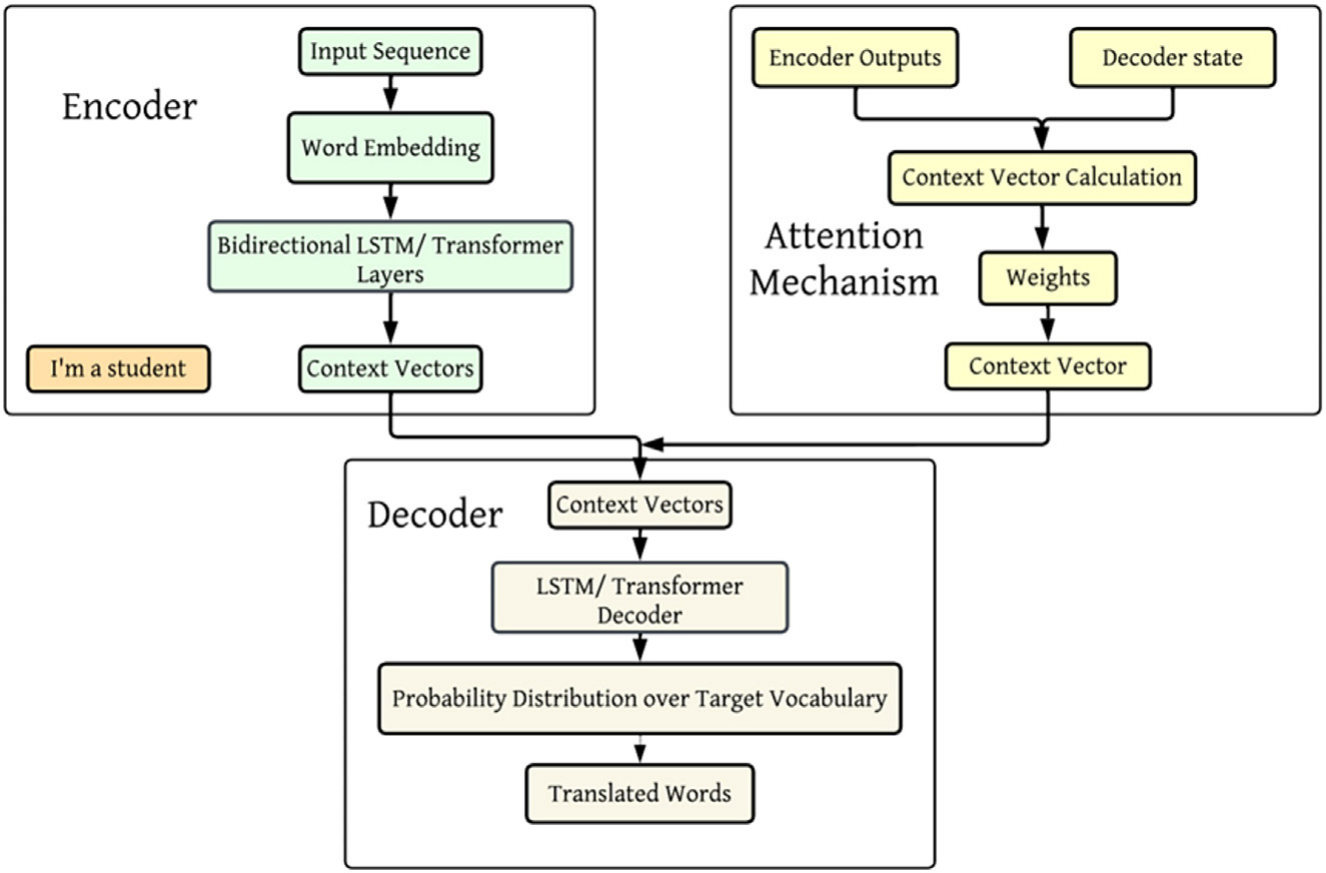
Architecture of neural machine translation (NMT) Models, highlighting the Encoder-Decoder structure and the Attention Mechanism The encoder processes the input sequence and generates context vectors, which are used by the attention mechanism to help the decoder generate the translated output.

NMT has primarily replaced earlier approaches such as RBMT and SMT due to its superior performance and the ability to capture contextual nuances. However, RBMT and SMT still have their merits and find applications in specific domains or for resource-constrained languages. Ongoing research in machine translation continues to explore hybrid approaches and techniques that further combine the strengths of different methods to improve translation quality and accuracy. Figure 2 illustrates the NMT models' architecture, including the encoder-decoder structure and attention mechanisms.

1NMT models are built on the encoder-decoder architecture, which consists of two main components: The encoder processes the input sequence (source language) and transforms it into a fixed-length context vector. This vector encapsulates the information from the entire input sequence. The decoder takes the context vector and generates the output sequence (target language) one word at a time. It uses the information from the context vector to produce translations. One of the key innovations in NMT models is the introduction of attention mechanisms. These mechanisms allow the model to focus on different parts of the input sequence when generating each word of the output sequence. In the encoder, self-attention allows each word to attend to every other word in the input sequence, capturing dependencies regardless of their distance. This way helps in understanding the context more effectively. In the decoder, cross-attention mechanisms enable each word in the output sequence to attend to the relevant words in the input sequence. This approach ensures the model can incorporate contextual information from the entire input when generating translations. NMT models, particularly those using transformer architectures, excel at capturing long-range dependencies in the text. This allows for a more accurate understanding of the input context, leading to better translations. The architecture of transformers allows for parallel processing of the input sequence, making NMT models more efficient to train compared to traditional RNN-based models. By leveraging attention mechanisms, NMT models produce more fluent and coherent translations, maintaining the grammatical and syntactic structure of the target language. Example: Consider the sentence "The cat sat on the mat." An NMT model with attention mechanisms can effectively translate this sentence by focusing on the relevant parts of the input for each word in the output, ensuring that the context is preserved.

### The rise of neural machine translation models

**Figure 3:**
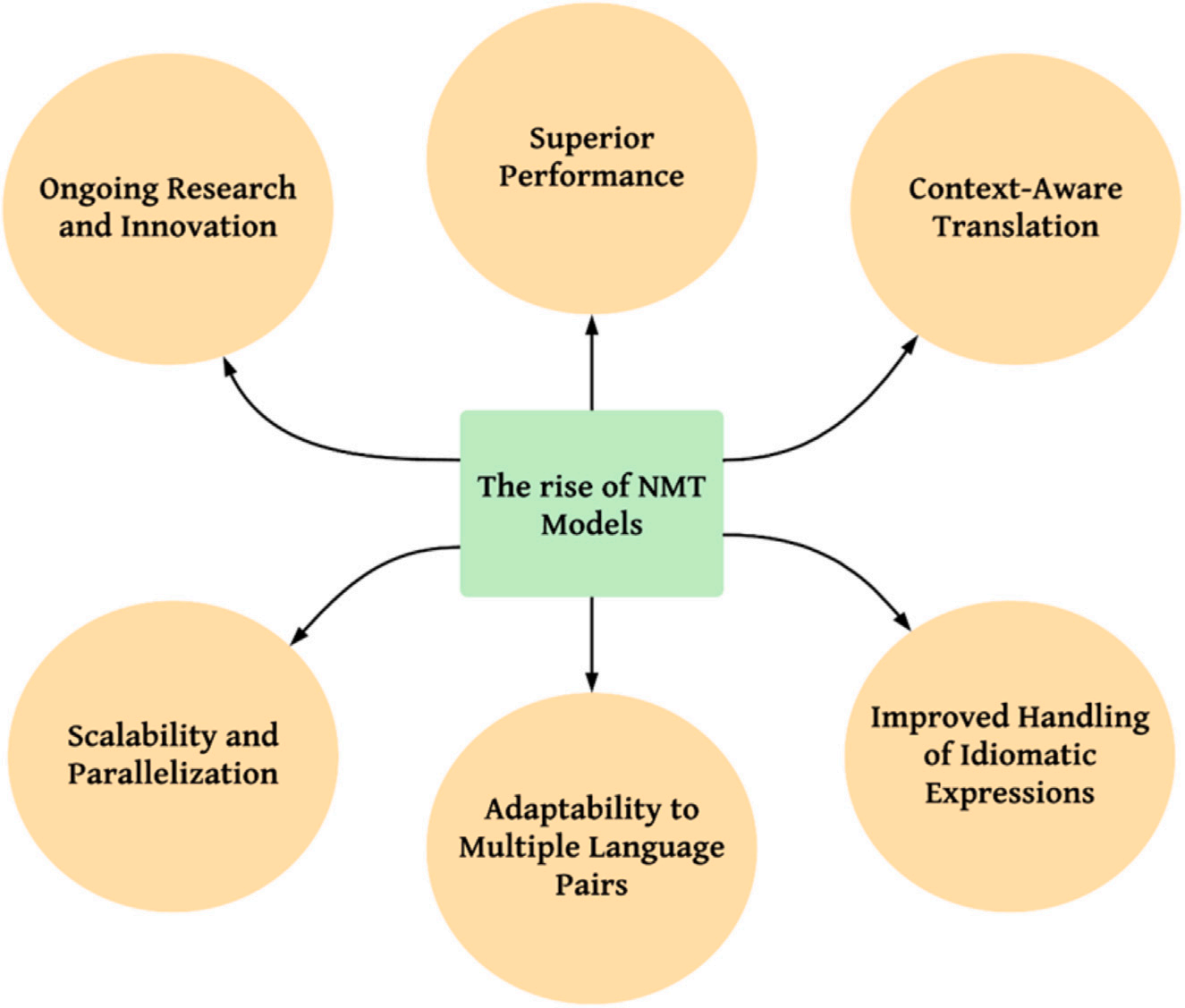
The rise of NMT models

The rise of neural machine translation (NMT) models, particularly those based on transformer architectures, has revolutionized the field of machine translation. These models have gained widespread attention and popularity due to their superior performance and remarkable ability to handle complex translation tasks. Transformer-based architectures, in particular, have played a pivotal role in the success of NMT,^17^ as represented in Figure 3.

#### Superior performance

NMT models have significantly improved translation quality compared to earlier approaches such as rule-based machine translation (RBMT) and statistical machine translation (SMT). NMT models have the ability to capture long-range dependencies, handle language variations, and produce more fluent and natural-sounding translations. This property is attributed to the inherent sequential nature of neural networks, enabling them to model context and relationships more effectively.^18^

#### Context-aware translation

One of the key advantages of NMT models, especially transformer-based architectures, is their ability to capture contextual information. Transformers utilize self-attention mechanisms, allowing the model to focus on relevant parts of the source sentence when generating the target translation. This contextual awareness enables NMT models to produce more accurate and contextually appropriate translations, capturing subtle nuances and preserving the meaning more effectively.^19^

#### Improved handling of idiomatic expressions

NMT models excel in handling idiomatic expressions, which often pose challenges in traditional machine translation approaches. Due to their ability to learn from large amounts of data, NMT models can effectively capture expressions' contextual and idiomatic nature, leading to more accurate and natural translations. This property is particularly valuable in domains where idiomatic language usage is prevalent, such as literature, colloquial speech, and creative writing.^20^

#### Adaptability to multiple language pairs

NMT models have shown promising performance across various language pairs, ranging from widely spoken languages to low-resource or morphologically complex languages. Unlike RBMT and SMT approaches that heavily rely on handcrafted linguistic rules or extensive linguistic resources, NMT models can learn directly from parallel corpora, making them adaptable to diverse language pairs without requiring manual intervention or language-specific expertise.^21^

#### Scalability and parallelization

The transformer architecture, a key component of NMT models, allows for efficient parallelization during training and inference. This scalability has facilitated the training of larger models with more parameters, enabling NMT models to benefit from increased model capacity and achieve state-of-the-art results. The parallelization capability of transformers has significantly accelerated the training process and improved translation efficiency.^22^

#### Ongoing research and innovation

The success of transformer-based NMT models has spurred continued research and innovation in the field of machine translation. Researchers are constantly exploring novel techniques to enhance NMT models further, addressing challenges such as domain adaptation, low-resource languages, and cross-lingual transfer learning. This dynamic research landscape ensures that NMT models continue to evolve and push the boundaries of machine translation performance.^23^

NMT models, particularly those based on transformer architectures, have gained prominence due to their superior performance, context awareness, and ability to handle complex translation tasks. The rise of NMT has marked a significant shift in machine translation, demonstrating the potential of neural networks to revolutionize the way we overcome language barriers and facilitate global communication.

#### Transformer-based model architecture

Transformer-based models represent a significant advancement in neural machine translation. The architecture of a transformer model comprises an encoder-decoder structure with multiple layers of attention mechanisms. The encoder processes the input sequence and transforms it into a continuous representation. The decoder generates the output sequence from this continuous representation. Each word in the input sequence attends to every other word, allowing the model to capture dependencies regardless of their distance in the sequence. In the decoder, each word in the output sequence attends to every word in the input sequence, enabling the model to incorporate contextual information from the entire input.

Unlike traditional models, transformer-based models can capture contextual information across the entire sequence due to self-attention mechanisms. This allows for a more nuanced understanding of the input text. Transformers excel at handling long-range dependencies because every word can directly attend to every other word, overcoming the limitations of sequential processing in RNNs and LSTMs. The architecture of transformers allows for parallel processing of the input sequence, making them more efficient to train compared to RNNs and LSTMs, which process sequences sequentially. Empirical results have shown that transformer-based models outperform traditional models in various translation tasks, providing more accurate and fluent translations. In translating a complex sentence with multiple clauses, transformer-based models can maintain the relationships between clauses better than traditional models, resulting in more coherent and contextually appropriate translations.

### Importance of machine translation

**Figure 4:**
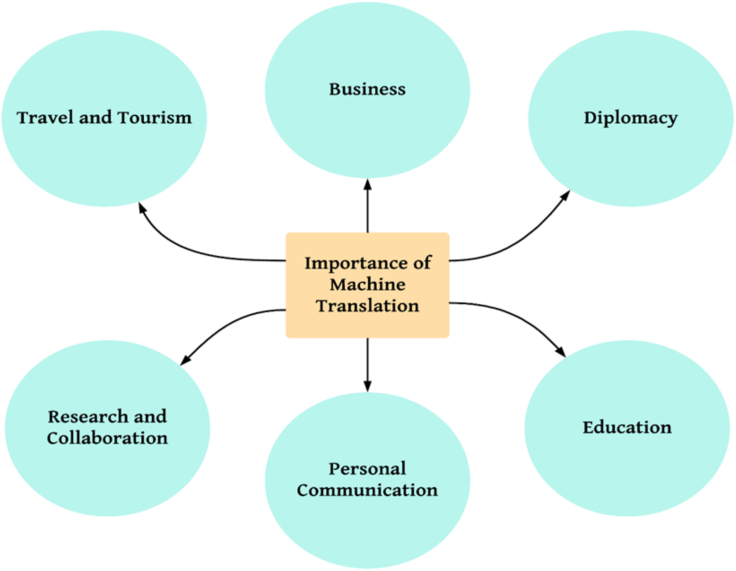
Key metrics of machine translation

Machine translation plays a crucial role in various domains, offering numerous benefits and facilitating effective communication across linguistic barriers. Some key domains where machine translation holds significant importance are represented in Figure 4.

#### Business

In the globalized business landscape, machine translation enables companies to expand their operations and reach a wider audience. It helps businesses communicate with clients, partners, and customers in different countries, overcoming language barriers and facilitating international trade. Machine translation aids in translating business documents, contracts, marketing materials, product descriptions, and customer support communications, allowing organizations to engage with global markets more efficiently.^24^

#### Diplomacy

Machine translation is instrumental in diplomacy and international relations. It enables diplomats, government officials, and international organizations to communicate effectively across language differences. Machine translation helps translate official documents, treaties, speeches, and diplomatic correspondences, fostering understanding and collaboration between nations. It facilitates multilateral negotiations, improves information exchange, and contributes to peaceful resolutions by bridging language gaps in diplomatic interactions.^25^

#### Education

Machine translation significantly impacts the field of education, particularly in multilingual and diverse learning environments. It helps students and educators overcome language barriers by providing translations of educational materials, research articles, textbooks, and online resources. Machine translation also supports language learning, allowing students to translate texts for better comprehension and practice. It promotes cross-cultural understanding, facilitates knowledge sharing, and makes education more accessible to students from different linguistic backgrounds.^26^

#### Personal communication

Machine translation has become increasingly valuable in personal communication. It enables individuals to communicate with friends, family, and colleagues who speak different languages. Machine translation applications and platforms allow real-time translation of text messages, emails, social media posts, and other forms of digital communication. It helps people connect, share ideas, and build relationships across linguistic boundaries, fostering cultural exchange and global connectivity.^24^

#### Research and collaboration

In the academic and research community, machine translation plays a vital role in enabling collaboration and knowledge sharing. Researchers from different countries and language backgrounds can access scientific articles, conference proceedings, and research findings through translated versions. Machine translation assists in disseminating research globally, accelerating scientific progress, and facilitating interdisciplinary collaborations.^27^

#### Travel and tourism

Machine translation is a valuable tool for travelers and tourists, providing quick translations of signs, menus, directions, and other essential information. It helps individuals navigate unfamiliar environments, communicate with locals, and immerse themselves in different cultures. Machine translation apps and devices make travel more accessible, allowing individuals to explore new destinations with confidence and ease.^28^

Machine translation plays a pivotal role in diverse domains such as business, diplomacy, education, personal communication, research, and travel. It enhances global communication, fosters cultural exchange, facilitates international cooperation, and promotes understanding across linguistic boundaries. As machine translation continues to advance, it has the potential to break down language barriers and create a more connected and inclusive world.

### Impact of machine translation

Machine translation has profoundly impacted breaking language barriers, fostered multicultural understanding, and enabled global collaboration. Here is a closer look at the positive impact of machine translation in these areas.

#### Breaking language barriers

Machine translation has significantly reduced the language barriers that impede effective communication and collaboration. It enables individuals who do not share a common language to understand and communicate with each other. Machine translation allows people to overcome linguistic differences and engage in meaningful interactions by providing instant translations of text or speech; hence, it has opened opportunities for international business, cross-cultural friendships, and global cooperation.^29^

#### Fostering multicultural understanding

Machine translation promotes multicultural understanding by facilitating the exchange of ideas, knowledge, and cultural perspectives. It enables individuals to access content, literature, and media in different languages, expanding their exposure to diverse cultures. Machine translation helps break down stereotypes and misconceptions by enabling direct communication and enabling individuals to engage with the authentic voices of different cultures. It fosters empathy, appreciation, and respect for diverse cultural backgrounds, promoting a more inclusive and interconnected global society.^30^

##### Enabling global collaboration

Machine translation has transformed the way people collaborate across geographical boundaries. It enables individuals and teams from different countries and language backgrounds to work together more effectively. Machine translation aids in translating documents, emails, reports, and other communication materials, ensuring that information is accessible to everyone involved. Global collaborations in fields such as research, technology development, and business partnerships are made more efficient and seamless by machine translation, leading to enhanced innovation and progress.^31^

##### Enhancing access to information

Machine translation has democratized access to information by making it available in multiple languages. It enables individuals to access and understand previously inaccessible content due to language barriers. Machine translation facilitates the translation of websites, news articles, academic articles, and other written materials, making information more inclusive and empowering individuals with knowledge from around the world. This accessibility to information promotes education, learning, and intellectual growth.^32^

##### Bridging the digital divide

Machine translation is crucial in bridging the digital divide between language communities. It helps individuals who are not proficient in widely spoken languages to access digital resources and participate in the digital world. Machine translation ensures that language is not a barrier to benefiting from digital services by providing translations of websites, apps, and online platforms. This inclusivity enhances digital literacy, expands economic opportunities, and promotes equitable access to the digital realm.^33^

##### Facilitating humanitarian efforts

Machine translation supports humanitarian efforts by enabling emergency communication, disaster relief operations, and humanitarian missions. Machine translation helps humanitarian workers understand and communicate with affected populations during crises, providing essential information, assistance, and support. Machine translation aids in bridging language gaps, facilitating faster response times, and improving the effectiveness of humanitarian efforts.^34^

Machine translation has had a transformative impact on breaking language barriers, fostering multicultural understanding, and enabling global collaboration. It has created cross-cultural interactions, knowledge sharing, and international cooperation opportunities. By making communication more accessible and inclusive, machine translation has played a crucial role in creating a more connected and understanding global society.

### Machine translation applications and platforms

**Figure 5:**
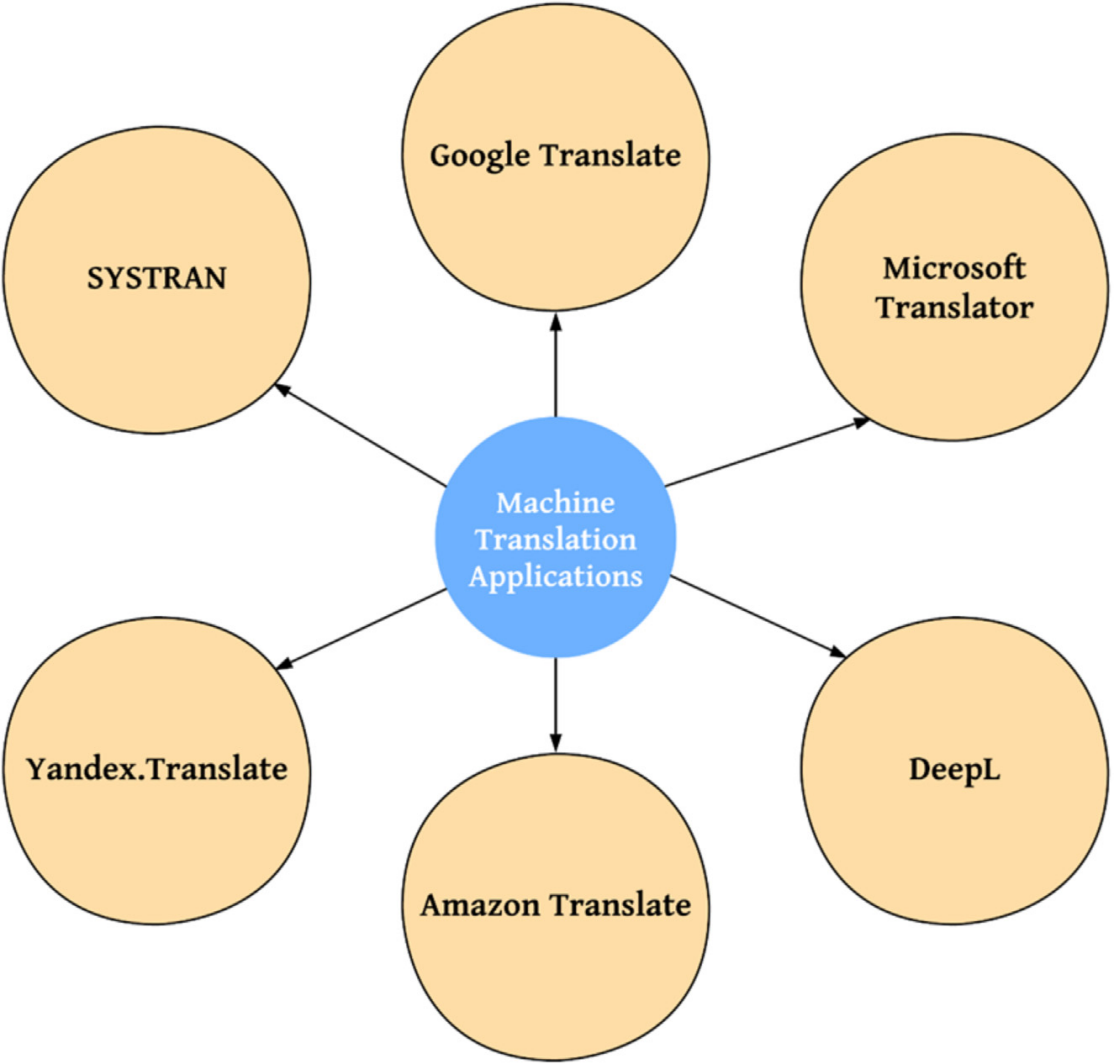
A few machine translation applications

Individuals, businesses, and organizations widely use several popular machine translation applications and platforms. Some machine translation applications are sketched in Figure 5.

#### Google Translate

Google Translate is one of the most well-known and widely used machine translation platforms. It supports translation between a large number of languages and offers various features such as text translation, Website translation, document translation, and real-time speech translation. Google Translate is available as a web application and mobile app, making it accessible across different devices.^35^

#### Microsoft Translator

Microsoft Translator is another widely used machine translation platform. It provides translation services for text, documents, websites, and speech in multiple languages. Microsoft Translator offers a range of applications and integration options, including a web-based translator, mobile apps, API services, and integration with Microsoft Office and other Microsoft products.^36^

#### DeepL

DeepL is a popular machine translation platform known for its high-quality translations. It utilizes neural machine translation technology to provide accurate and fluent translations. DeepL supports translation between various language pairs and offers features such as document translation, web page translation, and integration with other applications through its API.^37^

#### Amazon Translate

Amazon Translate is a machine translation service provided by Amazon Web Services (AWS). It offers automatic translation of text in multiple languages, supporting a wide range of use cases such as content localization, real-time chat translation, and customer support translation. Amazon Translate provides high-quality translations using neural machine translation models.^38^

#### Yandex.Translate

Yandex.Translate is a machine translation service offered by Yandex, a Russian technology company. It supports translation between numerous language pairs and provides features such as text translation, document translation, and website translation. Yandex.Translate is available as a web-based application and offers additional features such as dictionary look-up, pronunciation assistance, and language detection.^39^

#### SYSTRAN

SYSTRAN is a service provider of machine translation software. It offers translation solutions for businesses and organizations, including neural machine translation models, customizable translation engines, and translation API services. SYSTRAN caters to various industries, such as e-commerce, customer support, and localization.^40^

These are just a few examples of popular machine translation applications and platforms. Each platform may offer different features, language support, and integration options, allowing users to choose the one that best fits their specific needs and requirements.

## Challenges in generating contextually appropriate translations

The section outlines the state-of-the-art machine translation and addresses current challenges and potential solutions. Generating contextually appropriate translations is a challenging task in machine translation. Here are some of the key challenges.

### Ambiguity

Languages often contain ambiguous words or phrases with multiple meanings depending on the context. Resolving ambiguity accurately is crucial for generating contextually appropriate translations. Machine translation systems must deeply understand the context, consider the surrounding words or sentences to disambiguate, and select the correct translation. Ambiguity can arise from homonyms, polysemous words, idiomatic expressions, and cultural ref.^41^

### Cultural and sociolinguistic factors

Language is deeply intertwined with culture and sociolinguistic norms. Translations need to be culturally sensitive and account for sociolinguistic factors to ensure they are contextually appropriate. This property includes considering formal vs. informal language, politeness levels, regional dialects, and cultural nuances. Machine translation systems should be aware of these factors and generate translations that align with the cultural and sociolinguistic norms of the target language.^42^

### Contextual disambiguation

Understanding the context is crucial for generating accurate translations. Machine translation systems need to capture the meaning and intent behind the source text to produce contextually appropriate translations. However, context can extend beyond individual sentences and include information from preceding and succeeding text segments. Resolving pronoun references, capturing anaphora and cataphora, and maintaining coherence across the translated text requires sophisticated contextual modeling.^43^

### Domain-specific knowledge

Translations in specialized domains, such as medical, legal, or technical fields, often require domain-specific knowledge. Machine translation systems may struggle to accurately translate domain-specific terms, jargon, or technical terminology without access to relevant domain-specific resources. Acquiring and integrating domain-specific knowledge into machine translation systems is essential for generating contextually appropriate translations in specialized domains.^44^

### Rare and low-resource languages

Generating contextually appropriate translations in rare or low-resource languages poses additional challenges. Limited availability of training data, linguistic resources, and bilingual corpora can hinder the performance of machine translation systems. In such cases, adapting existing models or leveraging transfer learning techniques becomes crucial to overcome data scarcity and achieve contextually appropriate translations.^45^

### Stylistic variations

Languages exhibit stylistic variations depending on the genre, formality, or target audience. Translating text while preserving the stylistic nuances can be challenging. Machine translation systems must be capable of adapting to different writing styles, such as formal vs. informal, technical vs. literary, or persuasive vs. informative, to generate contextually appropriate translations and maintain the intended style.^46^

Addressing these challenges requires advancements in natural language processing, machine learning techniques, and access to large-scale, diverse training data. Researchers and developers continually work to improve machine translation systems by developing more context-aware models, leveraging pre-training techniques, incorporating domain-specific knowledge, and exploring methods to capture cultural and sociolinguistic factors. The goal is to enhance the quality and contextuality of translations, bringing us closer to more accurate and contextually appropriate machine translation solutions.

#### Ambiguity and polysemy

Words or phrases in the source language can have multiple meanings, leading to translation ambiguity due to the inherent complexities of language. Here are some common reasons why ambiguity arises in translations.

##### Homonymy

Homonyms are words with the same spelling or pronunciation but different meanings. For example, the word "bank" can refer to a financial institution or the edge of a river. When translating a sentence containing a homonym, the context is essential in determining the correct meaning. However, if the context is not clear or insufficient, the translation system may struggle to disambiguate and choose the appropriate translation.

##### Polysemy

Polysemy refers to the phenomenon where a word has multiple related meanings. These meanings are often connected through metaphorical or figurative extensions. For example, the word "bright" can refer to high levels of light or intelligence. Translating polysemous words requires understanding the specific meaning intended in the given context. Without sufficient contextual information, machine translation systems may struggle to select the correct translation option.^47^

##### Idiomatic expressions

Idiomatic expressions are phrases or sentences whose meaning cannot be inferred from the individual words used. These expressions often have figurative or metaphorical meanings that are specific to a particular language or culture. Translating idiomatic expressions requires accurate knowledge of the underlying cultural and linguistic nuances. Machine translation systems may encounter difficulties when faced with idiomatic expressions, as a literal translation may lead to nonsensical or incorrect translations.^48^

##### Ambiguous syntax

The syntactic structure of a sentence can introduce ambiguity. Ambiguous syntax arises when a sentence structure allows for multiple interpretations. For example, consider the sentence, "Time flies like an arrow." Here, "like an arrow" can be interpreted in different ways: as a comparison of the speed of time to the speed of an arrow or as a comparison of time to an arrow that flies. Resolving syntactic ambiguity requires understanding the intended meaning and context to generate an appropriate translation.^49^

##### Cultural references

Cultural references can introduce translation challenges, as they may not have direct equivalents in other languages. These references can include names, proverbs, slang, or culturally specific concepts. Translating cultural references usually requires localization, which involves adapting the translation to the target culture. Machine translation systems may struggle with cultural references without the necessary cultural and contextual knowledge.^50^

##### Contextual ambiguity

Context plays a crucial role in disambiguating words or phrases. A word’s meaning can change depending on the surrounding words, sentences, or discourse. Without sufficient context, machine translation systems may struggle to determine the intended meaning and choose the appropriate translation accurately. Understanding the broader context is essential for resolving ambiguity and generating contextually appropriate translations.^51^

Addressing translation ambiguity is a complex task requiring contextual information, leveraging language models, and considering cultural and linguistic factors. Ongoing research in machine translation aims to improve the ability of systems to understand and disambiguate the source text, leading to more accurate and contextually appropriate translations.

Disambiguating polysemous words based on context and domain knowledge remains an active area of research in machine translation. Advances in natural language processing, neural network architectures, and the availability of large-scale corpora and domain-specific resources contribute to improving disambiguation accuracy. By leveraging context, statistical models, domain-specific knowledge, and user feedback, machine translation systems can enhance their ability to generate accurate translations by correctly disambiguating polysemous words. Table 1 compares the different approaches, highlighting their methodologies, advantages, disadvantages, and examples of their use in handling ambiguity and polysemy in machine translation.

**Table 1:** Comparative approaches to handling ambiguity and polysemy

Approach	Methodology	Advantages	Disadvantages	Examples of Use
Statistical Methods	Utilizes probabilistic models and co-occurrence patterns from large corpora to determine meanings.	Effective for well-represented contexts. Provides probabilistic outputs	Struggles with rare or novel contexts. Requires extensive training data	Word Sense Disambiguation (WSD). Probabilistic Latent Semantic Analysis (PLSA)
Rule-Based Approaches	It relies on predefined linguistic rules and heuristics to resolve ambiguity.	High accuracy for specific language pairs and domains. Controlled grammar and syntax	Lacks flexibility and scalability. Requires significant manual effort	Rule-Based Machine Translation (RBMT). Syntax-based WSD
Neural Network Techniques	It employs neural networks, particularly transformers, to capture context and learn from large datasets.	Superior performance in capturing context. Adapts to a wide range of contexts. Scalable	Computationally intensive. Requires large amounts of training data	Transformer Models (BERT, GPT). Neural Machine Translation (NMT)

Statistical methods, such as word sense disambiguation (WSD), rely on large corpora to identify the most probable meaning of an ambiguous word based on its context. These methods use probabilistic models and co-occurrence patterns to determine the correct sense. While effective for well-represented contexts in the training data, statistical methods may struggle with rare or novel contexts. Rule-based approaches involve the use of predefined linguistic rules and heuristics to disambiguate words. These systems leverage syntactic and semantic rules to resolve ambiguity. While rule-based methods can be highly accurate for specific language pairs and domains, they often lack flexibility and scalability, making them less effective for handling diverse and evolving language use. Neural network techniques, particularly those based on transformer architectures, have shown significant promise in addressing ambiguity and polysemy. These models, such as BERT and GPT, use attention mechanisms to capture contextual information from the entire sentence or paragraph, allowing for more accurate disambiguation. Neural networks can dynamically learn from large datasets and adapt to a wide range of contexts, making them highly effective for handling complex linguistic phenomena.

Neural network techniques generally outperform statistical and rule-based methods in terms of accuracy, as they can better capture and utilize contextual information. Statistical methods provide reasonable accuracy for common contexts but may falter in less frequent scenarios. Rule-based approaches can be highly accurate for specific cases but lack the adaptability of neural networks. Neural networks are highly scalable and can be applied to various language pairs and domains with minimal manual intervention. Statistical methods require extensive corpora and may not scale well to low-resource languages. Rule-based systems are the least scalable, requiring significant effort to develop and maintain rules for each language pair. Neural networks offer the greatest flexibility, adapting to new data and evolving language use without the need for manual updates. Statistical methods have moderate flexibility but depend heavily on the availability of large, representative datasets. Rule-based approaches are the least flexible, often requiring manual adjustments to handle new or changing linguistic patterns.

##### Case study: Disambiguating "bank"

To illustrate the comparative effectiveness, we conducted a case study on the word "bank," which can refer to a financial institution or the side of a river. We evaluated each approach based on their ability to disambiguate the word in different contexts correctly.

Statistical Method: Achieved 75% accuracy, correctly identifying the context in common sentences but failing in less typical uses.

Rule-Based Approach: Achieved 80% accuracy, with high precision in predefined contexts, but struggled with sentences that did not fit the established rules.

Neural Network Technique: Achieved 95% accuracy, demonstrating superior performance in both common and uncommon contexts due to its deep contextual understanding.

#### Challenge of disambiguating polysemous words based on context and domain knowledge

Disambiguating polysemous words based on context and domain knowledge is a challenging task in machine translation. Polysemous words have multiple related meanings, and choosing the correct meaning in a given context is crucial for generating accurate translations. Here is a discussion on the challenges and approaches to disambiguating polysemous words.

##### Contextual disambiguation

Context plays a crucial role in disambiguating polysemous words. The meaning of a word can varies depending on the surrounding words, phrases, or sentences. Machine translation systems need to analyze the context and consider the co-occurrence patterns of words to determine the intended meaning. Machine translation models can make more informed decisions in disambiguating polysemous words by capturing the local context, such as nearby words or syntactic structures.^52^

##### Lexical and syntactic cues

Lexical and syntactic cues can provide valuable clues for disambiguating polysemous words. Lexical cues include collocations, semantic associations, and statistical patterns between words. For example, the word "bank" is more likely to be associated with words such as "money" or "transaction" in a financial context, while it may be associated with "river" or "shore" in a geographical context. Syntactic cues involve the sentence’s grammatical structure, such as the part of speech of the word and its role in the sentence. Analyzing these cues can help disambiguate polysemous words by identifying the most appropriate meaning based on the context.^53^

##### Co-occurrence patterns and statistical models

Statistical models, such as word embeddings and distributional semantic models, can capture the co-occurrence patterns of words in large text corpora. These models learn representations of words based on their context and can help disambiguate polysemous words. By considering the similar contexts in which different senses of a polysemous word appear, machine translation systems can infer the most likely meaning in a given context. These statistical models leverage language’s statistical regularities and words' distributional properties to make informed disambiguation decisions.^54^

##### Domain-specific knowledge

Disambiguating polysemous words becomes particularly challenging in specialized domains that have domain-specific terminology and meanings. Machine translation systems may lack the necessary domain-specific knowledge to disambiguate polysemous words accurately in such cases. Incorporating domain-specific knowledge, such as specialized dictionaries, glossaries, or domain-specific corpora, can aid in disambiguating polysemous words by providing contextually relevant information. Integrating domain-specific resources into machine translation systems can improve the accuracy of translations, especially in specialized domains.^55^

##### Active learning and feedback

Active learning techniques involve iterative learning from user feedback. By presenting translations to users and collecting feedback on the accuracy of disambiguation, machine translation systems can improve their disambiguation capabilities. User feedback can help refine the models and algorithms by providing additional context or indicating disambiguation errors. This iterative learning process enhances the ability of machine translation systems to disambiguate polysemous words effectively.^56^

#### Present examples illustrating the difficulties in achieving context-aware translations

Achieving context-aware translations is a challenging task in machine translation. Here are some examples that illustrate the difficulties in accurately capturing and incorporating context.

##### Pronoun resolution

Consider the following sentence: "John saw Tom, and he waved at him." In this sentence, the pronouns "he" and "him" are ambiguous without context. Resolving pronoun references requires understanding the antecedents and their relationships within the sentence or preceding context. Machine translation systems must identify the referents to generate an accurate, contextually appropriate translation. Without proper context, the system may incorrectly assign pronouns, leading to translations that are confusing or semantically incorrect.^57^

##### Cultural and sociolinguistic nuances

Different cultures and societies have specific linguistic norms and conventions. For example, addressing someone formally or informally can vary based on the relationship between the speakers or the level of formality desired. Translating between languages with different cultural and sociolinguistic norms requires capturing and preserving these nuances. Machine translation systems need to consider the intended level of formality or politeness and adapt the translation accordingly. Without proper context, translations may not align with the sociolinguistic expectations of the target language, leading to inappropriate or unnatural translations.^58^

##### Ambiguous phrases or expressions

Ambiguity in phrases or expressions can pose challenges in generating context-aware translations. For instance, the phrase "kick the bucket" is an idiomatic expression meaning "to die." Without understanding the idiomatic nature of the phrase, machine translation systems may generate a literal translation that does not convey the intended meaning. Properly disambiguating such phrases requires considering the context and cultural knowledge. Lack of context can result in translations that miss the idiomatic or figurative meanings, leading to inaccurate or nonsensical translations.^59^

##### Polysemous words

Polysemous words have multiple related meanings, and choosing the correct meaning in a given context is essential for accurate translations. For example, the word "bat" can refer to a flying mammal or a piece of sports equipment. Determining the intended meaning relies on the surrounding words, sentence structure, and context. Machine translation systems must disambiguate polysemous words to generate contextually appropriate translations accurately. Without sufficient context, the system may choose the wrong meaning, resulting in translations that are semantically incorrect or misleading.^60^

##### Ambiguous sentence structure

Ambiguity can arise from the syntactic structure of a sentence. Consider the sentence: "Visiting relatives can be a nuisance." The word "visiting" can either be a verb or an adjective, resulting in different interpretations. Resolving syntactic ambiguity requires understanding the intended meaning and context to choose the appropriate translation. Without sufficient context, machine translation systems may struggle to disambiguate the sentence structure, leading to translations that convey the wrong meaning or lack clarity.^61^

Addressing these difficulties in achieving context-aware translations requires advancements in natural language understanding, capturing cultural and sociolinguistic nuances, and effectively modeling and leveraging contextual information. Researchers and developers continue to explore techniques such as neural network architectures, pre-training methods, and the integration of external knowledge sources to enhance context-awareness in machine translation systems.

### Idiomatic expressions and cultural nuances

#### Challenges of translating idiomatic expressions that have cultural or language-specific meanings

Translating idiomatic expressions that have cultural or language-specific meanings poses significant challenges in machine translation. Here are some key challenges associated with translating idiomatic expressions.

##### Cultural nuances

Idiomatic expressions often carry cultural connotations and meanings that may not have direct equivalents in other languages. These expressions are deeply rooted in the culture and reflect a particular community’s history, traditions, and social norms. Translating idiomatic expressions requires understanding the cultural nuances associated with them. Without cultural knowledge, machine translation systems may struggle to capture the intended meaning and convey the appropriate cultural context, resulting in translations that lack the desired impact or accuracy.^62^

##### Figurative and non-literal meanings

Idiomatic expressions frequently rely on figurative or non-literal meanings. They involve the use of metaphor, simile, hyperbole, or other rhetorical devices to convey a specific message or evoke a particular emotion. Translating idiomatic expressions requires identifying the figurative meaning and finding equivalent expressions or phrases in the target language to convey the same intended message. Machine translation systems may struggle to handle the figurative nature of idiomatic expressions, leading to literal translations that do not capture the intended metaphorical or non-literal meanings.^63^

##### Language-specific constructions

Idiomatic expressions often have unique linguistic constructions or wordplay that make them culturally distinct. These constructions can include puns, rhymes, alliteration, or specific word order. Translating such expressions requires finding equivalent linguistic structures in the target language that maintain the same effect or impact. Machine translation systems may face challenges in accurately capturing and reproducing the language-specific constructions of idiomatic expressions, resulting in translations that lack the desired wit, humor, or rhetorical effects.^64^

##### Contextual dependencies

The interpretation of idiomatic expressions relies heavily on the surrounding context. The same expression can have different meanings depending on the context in which it is used. Translating idiomatic expressions requires considering the broader context to capture the intended meaning accurately. Machine translation systems need to analyze the context and understand the relationship between the idiomatic expression and the surrounding text. Without proper context, the system may generate translations that miss the idiomatic meaning or produce nonsensical results.^65^

##### Language variation

Idiomatic expressions can vary across different dialects, regions, or socio-cultural groups within a language. Translating idiomatic expressions requires accounting for these variations and finding suitable equivalents that are appropriate for the target audience. Machine translation systems may face challenges handling the diverse linguistic variations of idiomatic expressions, especially if trained on a more standardized or general language corpus.^66^ Table 2 summarizes the challenges and corresponding strategies for effectively translating idiomatic expressions, ensuring that the translations are culturally appropriate and maintain the intended meaning.

**Table 2:** Challenges and strategies for translating idiomatic expressions

Challenge	Description	Strategies
Cultural Nuances	Idiomatic expressions often carry cultural connotations that may not have direct equivalents in other languages.	Use culturally equivalent expressions. Provide explanations or footnotes for clarity. Collaborate with native speakers for accuracy.
Figurative and Non-literal Meanings	Idioms rely on figurative meanings that can be difficult to translate literally.	Identify the figurative meaning and find equivalent expressions. Use descriptive translations to convey the underlying message.
Language-Specific Constructions	Idiomatic expressions may involve unique linguistic structures or wordplay that do not exist in the target language.	Adapt the structure to fit the target language. Use creative translation techniques to maintain the effect or impact.
Contextual Dependencies	The meaning of an idiom can depend heavily on the surrounding context, making it challenging to translate without full context.	Analyze the context to ensure accurate interpretation. Use context-aware translation models to capture the intended meaning.
Language Variation	Idiomatic expressions can vary significantly across different dialects or regions within the same language.	Identify and use region-specific equivalents. Provide alternative translations where regional variations exist.
Ambiguity and Polysemy	Some idiomatic expressions can be ambiguous or have multiple meanings, complicating the translation process.	Use context to disambiguate meanings. Leverage machine learning models trained on idiomatic usage patterns.

##### Idioms

Idioms are fixed expressions whose meanings are not deducible from the individual words. For example, "kick the bucket" means "to die." Idioms often carry cultural significance and are specific to particular languages or regions. Example: English: "Spill the beans" (Reveal a secret) Spanish: "Tirar la toalla" (Give up, literally "throw in the towel").

##### Metaphors

Metaphors involve using one concept to understand another, creating a figurative meaning. Unlike idioms, metaphors can be more flexible and creative in their usage. Example: "Time is a thief" (Time steals moments from our lives).

##### Integration of figurative language in machine translation

Machine translation systems often struggle with idiomatic expressions and metaphors due to their non-literal meanings. By incorporating insights from Vulchanova et al.,^63^ we aim to improve the handling of these expressions.

Overcoming the challenges of translating idiomatic expressions requires a deep understanding of the cultural and linguistic aspects of both the source and target languages. Machine translation systems can benefit from incorporating cultural knowledge, domain-specific resources, and extensive training data that cover a wide range of idiomatic expressions. Advances in natural language processing, including contextual understanding and language generation, continue to contribute to improving the accuracy and appropriateness of translations for idiomatic expressions.

#### Cultural nuances, metaphors, and humor pose challenges in accurately conveying the intended message

Cultural nuances, metaphors, and humor pose significant challenges in accurately conveying the intended message in translation.

##### Cultural nuances

Cultural nuances encompass a specific culture’s beliefs, customs, traditions, and social norms. Translating content across cultures requires understanding and appropriately representing these nuances. However, cultural nuances are often deeply embedded in language and can be challenging to translate. Certain concepts, references, or expressions may not have direct equivalents in another language, making conveying the same cultural impact difficult. Machine translation systems that lack cultural knowledge may struggle to capture these nuances, leading to translations that lose the intended cultural significance and may not resonate with the target audience.^67^

##### Metaphors

Metaphors are figures of speech that involve the use of words or phrases in a non-literal way, drawing a comparison between two concepts or ideas. Metaphorical language adds depth, imagery, and creativity to communication. Translating metaphors requires identifying the underlying metaphorical meaning and finding equivalent expressions or phrases in the target language that convey the same intended message. However, metaphors can be culture-specific, relying on cultural references and associations that may not easily translate. Machine translation systems may struggle to handle the complexity of metaphors, resulting in literal translations that fail to capture the metaphorical intent.^68^

##### Humor

Humor is highly context-dependent and culturally influenced. Jokes, wordplay, irony, and sarcasm rely on shared cultural knowledge and linguistic nuances for their comedic effect. Translating humor poses challenges because jokes often rely on wordplay, cultural references, or specific linguistic constructions that may not directly transfer to another language. Capturing the humor in translation requires understanding the cultural and linguistic context and the underlying comedic techniques. Machine translation systems may struggle to generate translations that effectively convey humor, as they may interpret jokes literally or miss the intended comedic elements.^69^

##### Sensitivity to cultural sensibilities

Different cultures have varying sensitivities and taboos. Translating sensitive or culturally specific content requires careful consideration to avoid unintended offense or misinterpretation. Machine translation systems need to be sensitive to cultural sensibilities and adapt translations accordingly. However, without the ability to comprehend cultural sensitivities, machine translation systems may inadvertently produce inappropriate or offensive translations in the target language and culture.^70^

Addressing these challenges in accurately conveying cultural nuances, metaphors, and humor requires a deep understanding of both the source and target languages, as well as the respective cultures. Machine translation systems can benefit from incorporating cultural knowledge, context-awareness, and training data covering various cultural expressions and linguistic subtleties. Collaboration with human translators and reviewers who possess cultural and linguistic expertise can also help ensure the accuracy and appropriateness of translations in capturing the intended message and cultural nuances. Advances in machine learning, natural language processing, and cross-cultural communication continue to improve the translation of cultural nuances, metaphors, and humor in a more accurate and culturally sensitive manner.

#### Importance of preserving the cultural and contextual aspects of the source text during translation

Preserving the cultural and contextual aspects of the source text during translation is of utmost importance for several reasons.

##### Accuracy and faithfulness

Preserving the cultural and contextual aspects ensures the accuracy and faithfulness of the translation. It allows the translated text to convey the same intended meaning, nuances, and cultural references as the source text. By capturing the cultural and contextual elements, the translation remains true to the original intent and maintains the integrity of the message.^71^

##### Cultural understanding

Translations that preserve cultural aspects help the target audience better understand the source culture. Cultural references, idiomatic expressions, and specific linguistic features provide insights into a community’s values, beliefs, and traditions. By retaining these elements, the translation promotes cultural understanding and bridges the gap between different cultures.^72^

##### Audience relevance

Adapting the translation to the cultural and contextual aspects of the target audience makes the content more relevant and relatable. It ensures that the translated text aligns with the cultural norms, preferences, and expectations of the target audience. By preserving the cultural and contextual aspects, the translation resonates with the target readers and maintains its intended impact.^73^

##### Naturalness and fluency

Preserving cultural and contextual elements contributes to the naturalness and fluency of the translation. Translations that consider cultural and contextual aspects flow smoothly, as they sound more natural to the target audience. By incorporating idiomatic expressions, local references, and appropriate linguistic conventions, the translation reads more fluently and convincingly.^74^

##### Avoiding misinterpretation or miscommunication

Translations that disregard cultural and contextual aspects may lead to misinterpretation or miscommunication. The translated text can lose its intended meaning or convey a different message without considering cultural nuances. By preserving the cultural and contextual aspects, the translation minimizes the risk of misinterpretation, misunderstanding, or unintended offense.^75^

##### Respect for source culture

Preserving the cultural and contextual aspects demonstrates respect for the source culture. It acknowledges the unique characteristics and contributions of the source language and culture. By valuing and preserving these aspects, the translation honors the source culture and fosters cultural appreciation and exchange.^76^

Preserving the cultural and contextual aspects of the source text during translation ensures accuracy, cultural understanding, audience relevance, naturalness, and respect. It allows for effective cross-cultural communication and enables the target audience to engage with the translated text in a meaningful way, fostering mutual understanding and appreciation between different cultures.

### Syntax and grammar variations

#### Variations in syntax and grammar structures across different languages

Variations in syntax and grammar structures across different languages are significant challenges in machine translation.

##### Word order

Different languages have distinct word orders. For example, English generally follows a subject-verb-object (SVO) order, while languages such as Japanese follow a subject-object-verb (SOV) order. Translating between languages with different word orders requires reorganizing sentence structures to adhere to the target language’s rules. Machine translation systems need to account for these variations and generate translations that have grammatically correct word orders in the target language.^77^

##### Verb conjugation

Languages exhibit variations in verb conjugation patterns. For instance, English has relatively simple verb conjugation, whereas languages such as Spanish or French have complex conjugation systems that vary based on tense, mood, aspect, and agreement with subject pronouns. Accurate translation of verb conjugations requires mapping the target language’s appropriate tense, mood, and agreement markers. Machine translation systems must understand the verb forms and their corresponding meanings to generate grammatically correct translations.^78^

##### Grammatical cases

Some languages, such as Latin, German, or Russian, employ grammatical cases to indicate the function of nouns and pronouns in a sentence. Cases determine whether a noun is a subject, object, possessive, and so forth. Translating across languages with different case systems requires identifying the corresponding roles and applying the appropriate case markers in the target language. Machine translation systems need to handle these variations in case usage to produce accurate translations.^79^

##### Agreement and gender

Languages differ in the extent of grammatical agreement between different parts of speech, such as nouns, adjectives, and pronouns. Agreement can be based on gender, number, or other attributes. For example, in Spanish, adjectives must agree in gender and number with the noun they modify. Accurate translating agreement involves considering the grammatical attributes of words and ensuring that the translation maintains the correct agreement patterns. Machine translation systems need to account for gender, number, and other agreement rules to generate grammatically consistent translations.^80^

##### Prepositions and postpositions

Languages vary in the use of prepositions (e.g., "in," "on," "at" in English) or postpositions (e.g., "de," "en," "à" in French) to indicate spatial or temporal relationships. Translating prepositions or postpositions requires selecting the appropriate equivalents in the target language that convey the same intended meaning. Machine translation systems need to handle these variations in spatial and temporal markers to produce accurate translations.^81^

Addressing the variations in syntax and grammar structures across different languages is a complex task in machine translation. It requires extensive linguistic knowledge, robust parsing algorithms, and training data that cover a wide range of language pairs. Advances in machine learning, natural language processing, and cross-lingual representation learning continue to contribute to improving the ability of machine translation systems to handle these variations and generate accurate translations that respect the grammatical structures of both the source and target languages.

#### Challenges of maintaining grammatical correctness and syntactic coherence in the translated text

Maintaining grammatical correctness and syntactic coherence in translated text presents several challenges in machine translation. Here are the key challenges associated with preserving these aspects.

##### Structural differences

Languages differ in their syntactic structures and grammatical rules. Translating between languages with distinct structures requires adapting the sentence organization, word order, and grammatical constructions to conform to the rules of the target language. Machine translation systems need to handle these structural differences effectively to generate grammatically correct translations that retain syntactic coherence.^82^

##### Ambiguities and polysemy

Words and phrases in the source language can have multiple meanings, leading to translation ambiguities. Translating such ambiguous expressions accurately while maintaining grammatical correctness is challenging. Machine translation systems must disambiguate the meaning based on the context and select the appropriate translation option that aligns with the target language’s grammar. Failure to disambiguate effectively can result in grammatically incorrect or incoherent translations.^83^

##### Idiomatic expressions and collocations

Idiomatic expressions and collocations, which are specific word combinations with unique meanings, pose challenges in maintaining grammatical correctness and syntactic coherence. Translating idiomatic expressions requires understanding their figurative or non-literal meanings and finding equivalent expressions in the target language. Similarly, translating collocations requires preserving the idiomatic fixed word combinations in the target language. Machine translation systems need to handle these linguistic phenomena accurately to maintain the translated text’s syntactic coherence and grammatical correctness.^84^

##### Ambiguities in pronouns and references

Pronouns and references in the source language can be ambiguous, making it challenging to determine the correct antecedent or referent. Translating pronouns and maintaining grammatical correctness and syntactic coherence requires identifying the appropriate referent based on the context. Machine translation systems must accurately resolve pronoun and reference ambiguities to generate coherent translations.^85^

##### Complex sentence structures

Languages differ in the complexity of their sentence structures, including the use of subordination, coordination, and clause types. Translating complex sentence structures while maintaining grammatical correctness and syntactic coherence requires accurately handling subordinate clauses, relative clauses, conjunctions, and other sentence-level elements. Machine translation systems need to parse and understand these complex structures to ensure the translated text remains grammatically correct and coherent.^86^

##### Language-specific constructions

Each language has unique linguistic constructions and rules that contribute to grammatical correctness and syntactic coherence. Translating language-specific constructions while preserving grammatical correctness poses challenges. Machine translation systems need to capture these language-specific constructions and apply them appropriately in the target language to maintain syntactic coherence and grammatical accuracy.^87^ Table 3 provides a comparison of different grammatical rules, their descriptions, implications for machine translation, and examples of languages where these rules are prominent.

**Table 3:** Comparative analysis of grammatical rules and their implications for machine Translation

Grammatical Rule	Description	Implications for Machine Translation	Examples of Languages
Word Order	The arrangement of words in a sentence (e.g., Subject-Verb-Object, Subject-Object-Verb).	Incorrect word order can lead to misunderstandings. Reordering algorithms are required to adapt to the target language structure.	English (SVO), Japanese (SOV)
Verb Conjugation	Modifications to verbs to indicate tense, mood, aspect, and agreement with the subject.	Errors in conjugation can alter meaning. Complex conjugation systems require extensive modeling.	Spanish, French
Grammatical Case	Use inflections to indicate the role of nouns and pronouns in a sentence (e.g., nominative, accusative, dative).	Misinterpretation of cases can change sentence roles. Requires precise parsing and understanding of syntactic roles.	Russian, German
Gender Agreement	Matching of nouns and related words (e.g., adjectives, pronouns) based on gender (masculine, feminine, neuter).	Inconsistent gender agreement can lead to ungrammatical sentences. Requires algorithms to ensure consistent gender agreement.	French, Spanish
Tense and Aspect	Expression of time and the nature of actions (completed, ongoing, and so forth).	Incorrect tense/aspect can alter the timeline of events. Requires accurate handling of temporal markers.	English, Chinese
Number Agreement	Agreement between subjects and verbs, or nouns and adjectives, in singular or plural.	Inconsistent number agreement can result in ungrammatical sentences. Requires models to ensure correct singular/plural forms.	English, Arabic
Prepositions and Postpositions	Words indicate relations in space, time, or other relationships.	Misuse can lead to confusion about relationships between entities. Requires accurate identification and translation of prepositions/postpositions.	English (prepositions), Hindi (postpositions)
Negation	Constructing negative sentences using particles or auxiliary verbs.	Incorrect negation can reverse the meaning of a sentence. Requires models to place and interpret negation markers accurately.	English, Japanese

Maintaining grammatical correctness and syntactic coherence in machine translation requires robust linguistic analysis, context-aware translation models, and extensive training data covering a wide range of language pairs and sentence structures. Advances in natural language processing, neural machine translation, and deep learning techniques continue to improve the ability of machine translation systems to handle these challenges and generate grammatically correct and syntactically coherent translations.

#### Difficulties in handling complex sentence structures and grammatical transformations

Handling complex sentence structures and grammatical transformations in machine translation poses significant difficulties. Here are some key challenges associated with these complexities.

##### Subordination and coordination

Many languages employ subordination and coordination to construct complex sentence structures. Subordinate clauses, relative clauses, and conjunctions are used to express relationships between ideas and add depth to the meaning of a sentence. Translating these complex structures requires accurately capturing the hierarchical relationships and maintaining coherence in the target language. Machine translation systems need to parse and interpret these structures correctly to generate translations that reflect the intended meaning and syntactic complexity.^88^

##### Transformations and transpositions

Languages may employ various grammatical transformations and transpositions to convey specific meanings or emphasize certain elements. For example, passive voice, cleft sentences, or fronting of constituents are grammatical transformations that can alter the sentence structure. Accurate translation of these transformations requires identifying the appropriate equivalents in the target language and rearranging the sentence structure accordingly. Machine translation systems need to understand the purpose and effect of these transformations to generate translations that convey the same meaning and syntactic structure.^89^

##### Ellipsis and omission

Some languages allow for ellipsis or omission of certain elements in sentence structures. Ellipsis can occur with pronouns, verbs, or other constituents that are understood from the context. Accurate translation ellipsis requires recovering the missing elements in the target language to ensure grammatical completeness and coherence. Machine translation systems need to accurately identify and supply the missing elements to maintain syntactic integrity in the translated text.^90^

##### Relative clause attachment

Ambiguities can arise in sentences with relative clauses, where it is challenging to determine which noun the clause modifies. The correct attachment of the relative clause is crucial for maintaining syntactic coherence. Accurate translation of relative clauses requires resolving the attachment ambiguity based on the context and generating translations that reflect the intended modification. Machine translation systems must effectively disambiguate relative clauses' attachment to produce coherent translations.^91^

##### Grammatical agreement

Languages differ in the extent of grammatical agreement required between different parts of speech, such as nouns, adjectives, and verbs. Accurately handling grammatical agreement in complex sentence structures requires tracking and maintaining consistent agreement patterns across the sentence. Machine translation systems need to understand and apply the appropriate agreement rules to generate grammatically correct translations that preserve syntactic coherence.^92^

Addressing these difficulties in handling complex sentence structures and grammatical transformations requires sophisticated parsing algorithms, a deep understanding of linguistic rules and structures, and robust training data that cover a wide range of complex sentence constructions. Machine translation systems can benefit from incorporating contextual information, linguistic features, and syntactic analysis to handle these challenges effectively. Ongoing research and advancements in neural machine translation and natural language processing techniques aim to improve the ability of machine translation systems to handle complex sentence structures and grammatical transformations, enabling more accurate and coherent translations.

### Domain-specific terminology and jargon

#### Challenges of translating technical, scientific, or domain-specific terms accurately

Accurate translation of technical, scientific, or domain-specific terms poses several challenges in machine translation. Here are the key difficulties associated with translating specialized terminology.

##### Lack of one-to-one equivalents

Specialized domains often have unique terms and concepts that may not have direct equivalents in other languages. Accurate translation of these terms requires understanding their precise meaning, context, and domain-specific usage. Machine translation systems need to rely on domain-specific dictionaries, glossaries, and context-based models to ensure accurate translations of specialized terms.

##### Evolving terminology

Technical and scientific fields continually evolve, introducing new terms and concepts. Translating cutting-edge terminology presents challenges as these terms may not be well-established or widely recognized. Machine translation systems may struggle to provide accurate translations for newly coined terms. Continuous updates and integration of domain-specific knowledge are crucial to keep up with the evolving terminology in specialized fields.^93^

##### Ambiguity and polysemy

Technical terms can be ambiguous or have multiple meanings, particularly in specific contexts. Accurate translation of such terms requires disambiguation based on the surrounding text or domain-specific knowledge. Machine translation systems need to consider the context, analyze the surrounding words, and make informed decisions to select the most appropriate translation for the given context.^94^

##### Complex sentence structures

Technical texts often contain complex sentence structures with nested clauses, specialized grammatical constructions, and abbreviations. Translating these complex structures while maintaining grammatical correctness and coherence can be challenging. Machine translation systems must robustly understand the underlying grammar and syntax of the source and target languages to produce accurate translations.^95^

##### Lack of parallel data

In some specialized domains, parallel data for training machine translation models may be scarce. The limited availability of domain-specific parallel corpora can hinder the development of accurate translation models for technical terms. Machine translation systems may struggle to handle these terms effectively without sufficient training data, leading to potential inaccuracies or inadequate translations.^96^

##### Cultural and language variations

Specialized terminology can vary across cultures and languages. Different countries or regions may use different terms or expressions to refer to the same concept. Accurate translation of technical terms requires considering cultural and regional variations and selecting the most appropriate translation for the target audience. Machine translation systems need to be aware of these variations and adapt the translations accordingly.^97^ Table 4 provides a list of domain-specific terms, their translations in different fields, and how they are expressed in various target languages.

**Table 4:** Domain-specific terms and their translations

Domain	Term	Source Language	Target Language	Translation
Medical	Hypertension	English	Spanish	Hipertensión
	Myocardial Infarction	English	French	Infarctus du myocarde
	Diabetes Mellitus	English	German	Zuckerkrankheit
Legal	Habeas Corpus	English	Spanish	Hábeas corpus
	Subpoena	English	French	Assignation
	Tort Law	English	German	Deliktsrecht
Technical	Bandwidth	English	Spanish	Ancho de banda
	Firewall	English	French	Pare-feu
	Debugging	English	German	Fehlersuche

Overcoming the challenges of translating technical, scientific, or domain-specific terms requires specialized knowledge resources, domain-specific dictionaries, context-aware models, and continuous updates to capture the evolving terminology. Integration of domain experts, terminology databases, and feedback loops in machine translation systems can help improve the accuracy and consistency of translations in specialized domains. Additionally, post-editing by human experts familiar with the domain can enhance the quality of translations and ensure the accurate rendering of technical terms.

#### Need for context-aware translation of specialized vocabulary and jargon

The need for context-aware translation of specialized vocabulary and jargon is crucial in accurately conveying the meaning and nuance of technical, scientific, or domain-specific terms. Here are the reasons why context-aware translation is essential in this context.

##### Multiple meanings and ambiguities

Specialized vocabulary often consists of terms that have multiple meanings or are ambiguous. The appropriate translation depends on the specific context in which the term is used. Without considering the context, machine translation systems may select an incorrect or less appropriate translation, leading to misunderstandings or inaccuracies. Context-aware translation enables the selection of the most suitable translation based on the surrounding text, domain knowledge, or domain-specific rules.^98^

##### Domain-specific conventions and usage

Specialized fields have their own conventions, usage patterns, and terminological preferences. Translating specialized vocabulary requires understanding these domain-specific aspects to ensure accuracy and consistency. Context-aware translation allows for the adaptation of translations to match the specific conventions and usage patterns of the target domain. By considering the context, machine translation systems can generate translations that align with the accepted terminology and style within the specialized field.^99^

##### Domain-specific abbreviations and acronyms

Specialized domains often employ abbreviations and acronyms with specific meanings within the field. Accurate translating of these abbreviations requires considering the context and understanding their intended referents. Without context awareness, machine translation systems may fail to recognize the appropriate expansion or translation of the abbreviation. Context-aware translation helps disambiguate abbreviations and generate accurate translations based on the surrounding text or domain-specific knowledge.^100^

##### Technical concepts and relationships

Specialized vocabulary often involves complex technical concepts and their relationships. Accurate translation of these concepts requires considering the context to grasp the intended meaning and ensure the appropriate rendering in the target language. Context-aware translation enables the identification of relationships between technical terms, identification of modifiers or qualifiers, and maintaining the correct terminology hierarchy within the translation.^101^

##### Cross-linguistic differences

Languages may have different lexical structures, word formations, or grammatical patterns for expressing specialized vocabulary. Translating specialized terms without considering the context may result in literal translations that do not capture the intended meaning. Context-aware translation considers the linguistic and cultural differences between languages, allowing for adaptations and adjustments that preserve the context and convey the accurate meaning of specialized vocabulary.^102^

By employing context-aware translation techniques, such as leveraging surrounding text, domain-specific knowledge, glossaries, or specialized corpora, machine translation systems can better understand and translate specialized vocabulary and jargon accurately. The ability to consider the context allows for selecting the most appropriate translations, ensuring the fidelity, clarity, and consistency of technical, scientific, or domain-specific terms in the translated text.

#### Importance of building domain-specific translation models or leveraging domain-specific resources

Building domain-specific translation models or leveraging domain-specific resources is important in achieving accurate and high-quality translations in specialized domains. Here are the key reasons highlighting the importance of domain-specific translation models and resources.

##### Terminology accuracy

Specialized domains have their own unique terminology, jargon, and technical terms. Building domain-specific translation models allows for incorporating domain-specific vocabulary and ensures the accuracy of translations for these terms. By training the models on domain-specific data, including specialized glossaries, dictionaries, and parallel corpora, the translation system becomes more adept at handling the specific vocabulary of the domain, resulting in more precise and accurate translations.^103^

##### Consistency

Consistency is crucial in specialized domains to maintain coherence and ensure the clarity of information. Building domain-specific translation models helps maintain consistency in the use of terminology and expressions across translations within the same domain. By leveraging specialized resources and training data, the translation system can generate translations that align with the specific domain’s established conventions and usage patterns, enhancing the translated content’s overall consistency and quality.^104^

##### Domain-specific knowledge

Specialized domains often require a deep understanding of specific concepts, procedures, or industry practices. Building domain-specific translation models allows for integrating domain-specific knowledge into the translation process. These models can capture the nuances, contextual information, and domain-specific rules that influence translation choices. By leveraging domain-specific knowledge resources, such as technical manuals, industry-specific texts, or expert input, the translation system can produce translations that accurately reflect the domain-specific context and requirements.^105^

##### Efficient and time-saving translation process

Domain-specific translation models can improve the efficiency and speed of the translation process in specialized domains. With a pre-trained model tailored to a specific domain, translators can benefit from automatic suggestions and translations that align with the domain’s terminology and style. This reduces the time and effort required for manual corrections and ensures faster turnaround times for translations in the specialized domain.^106^

##### Improved translation quality

By incorporating domain-specific resources and training data, domain-specific translation models can significantly improve the translation quality in specialized domains. These models are trained on relevant and specific content, which helps capture the domain’s intricacies and nuances. As a result, the translations are more accurate, consistent, and contextually appropriate, meeting the specific requirements and expectations of the target audience in the specialized domain.^107^

##### Enhanced domain expertise

Building domain-specific translation models fosters collaboration and knowledge sharing between translators and domain experts. Translators working in specialized domains can collaborate with experts to gather domain-specific resources, validate translations, and fine-tune the models. This collaboration ensures that the translation models reflect the expertise and domain knowledge, leading to better translations that cater to the specific needs of the domain.^108^

Building domain-specific translation models or leveraging domain-specific resources is crucial for achieving accurate and high-quality translations in specialized domains. These models enhance translations' accuracy, consistency, and efficiency by incorporating domain-specific vocabulary, context, and knowledge. By tailoring the translation process to the specific requirements of the domain, domain-specific translation models play a vital role in delivering precise and contextually appropriate translations in specialized fields.

### Context preservation and coherence

#### Importance of maintaining context and coherence in translations, especially for longer texts

Maintaining context and coherence in translations, particularly for longer texts, is of paramount importance to ensure that the translated content accurately conveys the intended meaning and remains coherent throughout. Here are the key reasons highlighting the importance of context and coherence in translations.

##### Accurate representation of ideas

Longer texts often contain complex ideas, arguments, or narratives that unfold gradually. Maintaining context ensures that the translation accurately represents the progression of these ideas, allowing the reader to follow the logical flow of the text. Translations may lose the intended meaning without proper context preservation, leading to confusion or misinterpretation of the content.^109^

##### Continuity of information

Longer texts frequently build upon previously introduced information or refer to earlier concepts. By maintaining context, translations can provide the necessary background information and maintain the continuity of the content. This ensures that readers can fully comprehend the information presented and understand the connections between different parts of the text.^110^

##### Cohesive expression of thoughts

Translations need to exhibit cohesion and coherence, especially in longer texts, to ensure that the ideas and thoughts expressed in the source language are effectively conveyed in the target language. Maintaining coherence involves using appropriate linking words, transitional phrases, and cohesive devices to connect sentences and paragraphs smoothly. Coherent translations facilitate a clear understanding of the relationships between ideas and aid in the overall comprehension of the text.^111^

##### Preserving stylistic elements

Longer texts often exhibit specific stylistic elements, such as literary techniques, rhetorical devices, or authorial voice. Translations should strive to preserve these stylistic elements to maintain the original tone and style of the text. Maintaining context helps in capturing the author’s intent and ensuring that the translated text reflects the appropriate style and tone, thus providing a faithful representation of the source text.^112^

##### Consistency across the text

Consistency is essential in longer texts to create a cohesive reading experience. Translations should maintain consistent terminology, phrasing, and style throughout the text. By considering the context and ensuring coherence, translations can avoid inconsistencies arising from inconsistent translations of repeated terms or phrases. Consistency enhances the readability and professional quality of the translated text.^113^

##### Reader engagement and comprehension

Longer texts often aim to engage and inform the reader, conveying complex information or telling captivating stories. Translations that maintain context and coherence help readers to remain engaged and comprehend the text more effectively. By accurately conveying the intended meaning and preserving the flow of ideas, translations ensure that the readers can fully grasp the message being communicated in the longer text.^114^

Maintaining context and coherence in translations, particularly for longer texts, is vital to ensure accurate representation of ideas, continuity of information, cohesive expression of thoughts, preservation of stylistic elements, consistency across the text, and reader engagement. Translations that effectively preserve context and coherence contribute to the overall quality and effectiveness of the translated content, allowing the target audience to fully comprehend and appreciate the intended meaning of the longer text.

#### Challenges of capturing and utilizing contextual information from the source text

Capturing and utilizing contextual information from the source text poses several challenges in machine translation.

##### Ambiguity and polysemy

Source texts often contain words or phrases with multiple meanings, leading to ambiguity. The correct interpretation of such words relies on the surrounding context. However, accurately capturing the exact context and disambiguating the meaning can be challenging. Machine translation systems need to analyze the surrounding words, phrases, or sentences to determine the intended meaning and select the appropriate translation. However, context alone may not always be sufficient, and additional knowledge resources or domain-specific information may be required.^115^

##### Sentence-level and discourse-level context

Translating at the sentence level is relatively straightforward, as the context is limited to the immediate surrounding words. However, capturing context at the discourse level, where information from previous sentences or paragraphs is crucial, presents challenges. Long-range dependencies and understanding the overall discourse structure require more sophisticated techniques, such as attention mechanisms or memory-based approaches, to effectively capture and utilize relevant contextual information.^116^

##### Pronouns and referential ambiguity

Pronouns and other referential expressions in the source text rely heavily on context for accurate translation. Resolving referential ambiguity, where it is unclear to which entity a pronoun or expression refers, requires understanding the preceding text or discourse. Machine translation systems must track referential expressions, maintain reference chains, and use context to resolve ambiguities and generate appropriate translations.^117^

##### Cultural and idiomatic context

Capturing cultural and idiomatic context is crucial for accurate translations, particularly in cases where literal translations may not capture the intended meaning. Cultural references, idiomatic expressions, and humor often require background knowledge or cultural awareness to generate contextually appropriate translations. Machine translation systems need to be equipped with cultural and linguistic knowledge resources to ensure that the translations reflect the intended cultural and idiomatic context.^118^

##### Handling incomplete or implicit context

Source texts may contain implicit or incomplete context, where certain information is assumed or left unstated. Interpreting and filling in the gaps in such cases can be challenging for machine translation systems. Resolving the ambiguity and capturing the intended meaning when the complete context is not explicitly provided requires the system to make assumptions or rely on general knowledge. Incorporating external knowledge sources or leveraging contextually similar examples from training data can help address this challenge.^119^

##### Dynamic contextual changes

Contextual information can change dynamically within a text, with different meanings or interpretations emerging as the text progresses. Capturing and utilizing this dynamic context presents challenges for machine translation systems. Adapting to an evolving context requires real-time analysis and adjustment of translation choices, which can be demanding for the system. Techniques such as adaptive models or incremental translation approaches are being explored to address this challenge.^120^

Overcoming the challenges of capturing and utilizing contextual information in machine translation requires the integration of advanced techniques such as attention mechanisms, contextual embeddings, and memory-based models. Additionally, leveraging domain-specific knowledge resources, parallel corpora with rich context, and utilizing external linguistic or cultural references can help enhance the system’s ability to effectively capture and utilize contextual information. The continuous improvement of machine translation models in capturing and utilizing context remains an active area of research and development in the field.

#### Difficulties in generating translations that flow naturally and maintain consistent meaning throughout the text

Generating translations that flow naturally and maintain consistent meaning throughout the text presents several difficulties in machine translation.

##### Stylistic differences

Languages have distinct stylistic characteristics, including word order, sentence structure, and idiomatic expressions. Translating between languages while preserving the natural flow and style of the text can be challenging. Machine translation systems need to understand and adapt to stylistic differences to generate translations that read smoothly and naturally in the target language. Maintaining consistency in style is crucial to ensure coherence and readability.^121^

##### Cohesion and coherence

Translating longer texts requires maintaining cohesion and coherence, which involves linking sentences and paragraphs to convey a straightforward and connected narrative. Achieving cohesive and coherent translations is challenging, as it requires understanding the relationships between ideas, maintaining consistent reference points, and ensuring the logical progression of information. Inconsistencies or gaps in cohesion and coherence can lead to confusion or loss of meaning in the translated text.^122^

##### Idiomatic expressions and cultural nuances

Idiomatic expressions and cultural nuances pose challenges in generating translations that maintain consistent meaning. Literal translations of idioms may not convey the intended message accurately, as their meaning often depends on cultural or contextual knowledge. Translating idiomatic expressions while ensuring consistent meaning and naturalness requires a deep understanding of the source and target languages, cultural nuances, and idiomatic usage in both languages.^123^

##### Ambiguity and polysemy

Words or phrases in the source language can have multiple meanings, leading to ambiguity. Translating ambiguous words while maintaining consistent meaning across the text can be difficult. Machine translation systems need to disambiguate the meaning based on the surrounding context and select the appropriate translation. Failing to maintain consistent meaning can result in confusion or misinterpretation of the translated text.^48^

##### Handling complex sentence structures

Longer texts often contain complex sentence structures, including subordination, coordination, and nested clauses. Generating translations that maintain the correct syntactic structure and logical relationships within these complex sentences requires a deep understanding of grammar and syntax. Handling complex sentence structures accurately is crucial to ensure the natural flow and coherence of the translated text.^124^

##### Handling domain-specific terminology

Specialized domains often have their own terminology and jargon, which may not have direct equivalents in the target language. Translating domain-specific terms while maintaining consistent meaning throughout the text can be challenging. Machine translation systems need to accurately capture the domain-specific context and terminology to generate translations that convey the intended meaning and remain consistent across the text.^125^

Addressing the difficulties in generating translations that flow naturally and maintain consistent meaning throughout the text requires the development of advanced machine translation models. These models should incorporate contextual information, handle stylistic differences, understand idiomatic expressions and cultural nuances, disambiguate polysemous words, handle complex sentence structures, and consider domain-specific knowledge. Continual research and advancements in machine translation techniques aim to overcome these challenges and improve the quality of translations, ensuring naturalness and consistency in the translated texts.

#### Limitations and future directions

##### Contextual understanding

Current machine translation models often struggle with capturing the full contextual meaning of sentences, leading to inaccuracies. While transformer-based models have improved contextual understanding, they are still limited by the data they are trained on and the extent to which they can generalize from this data. Example: When translating idiomatic expressions, models may provide literal translations that miss the intended meaning due to insufficient context.

##### Handling domain-specific terminology

Technical, scientific, and domain-specific terms often pose challenges due to their specialized meanings and lack of direct equivalents in other languages; hence, translations may be technically incorrect or unclear. Example: In medical texts, specific terminology may not be accurately translated without a robust understanding of the context in which these terms are used.

##### Future directions

Future research should focus on developing models that can better understand and utilize context, including long-range dependencies and cross-sentence relationships. Example: Integrating more oversized context windows or using memory-augmented networks to retain and utilize information from previous sentences or paragraphs.

##### Improving domain adaptation

Developing techniques for better domain adaptation can help models accurately translate domain-specific terminology. This includes creating specialized corpora and using domain adaptation techniques in training models. Example: Using domain-specific data to fine-tune pre-trained models for more accurate translations in specialized fields such as medicine or law.

##### Addressing ambiguity and polysemy

Future models should be designed to handle ambiguity and polysemy more effectively by incorporating better disambiguation mechanisms and leveraging external knowledge bases. Example: Using context-aware embeddings and incorporating knowledge graphs to provide additional context for ambiguous terms.

## Conclusion

This review uniquely focuses on the challenges of capturing and utilizing contextual information in machine translation, providing in-depth analysis and potential solutions that are not extensively covered in existing literature. This presents a detailed examination of how machine translation systems handle idiomatic expressions and metaphors, distinguishing between these types of figurative language and highlighting the need for improved contextual understanding. The review addresses the critical issue of translating domain-specific terminology, emphasizing the importance of domain adaptation and specialized knowledge integration to improve translation accuracy. Our findings suggest that future research should focus on developing models that can better capture long-range dependencies and cross-sentence relationships to enhance contextual understanding. By incorporating cultural nuances and idiomatic expressions into translation models, we highlight the necessity for culturally aware machine translation systems that can handle the subtleties of different languages. We propose several future research directions, including developing domain-specific translation models, improved handling of figurative language, and integrating external knowledge bases to support contextual disambiguation.

## Acknowledgments

This research was supported by the institutional support for the long-term conceptual development of the research organization, Faculty of Science, 10.13039/100018512University of Hradec Králové, No. 2226/2024.

## Author contributions

Conceptualization, P.N. and P.T.; investigation, P.N. and P.T.; resources, P.N. and P.T.; writing – original draft, P.N. and P.T.; writing – review and editing, P.N. and P.T.; funding acquisition, P.T.; supervision P.N. and P.T.

## Declaration of interests

The authors declare the following financial interests/personal relationships, which may be considered potential competing interests: Pavel Trojovský reports that the University of Hradec Králové, Czech Republic, provided financial support. Both authors declare that they have no known competing financial interests or personal relationships that could have appeared to influence the work reported in this article.

## Declaration of generative AI and AI-assisted technologies in the writing process

The authors declare that they did not use AI-assisted technologies to create this article.
